# Supplementary figures and images for: Endomembranes promote chromosome missegregation by ensheathing misaligned chromosomes
Source: J Cell Biol. 2022 Apr 29;221(6):e202203021. doi: 10.1083/jcb.202203021 (PMC9066052; doi:10.1083/jcb.202203021)

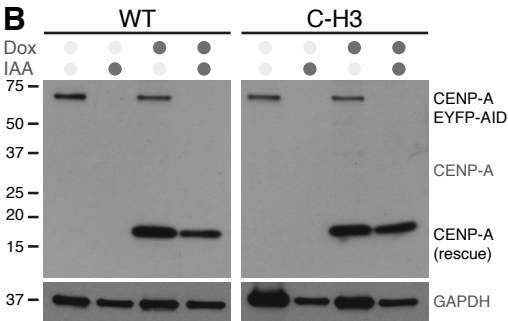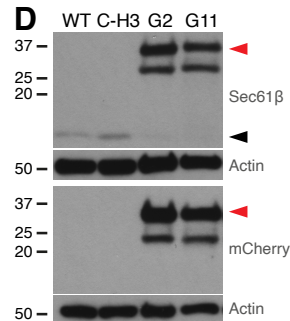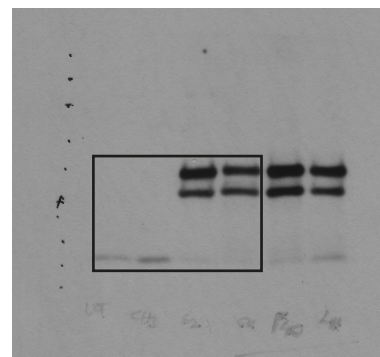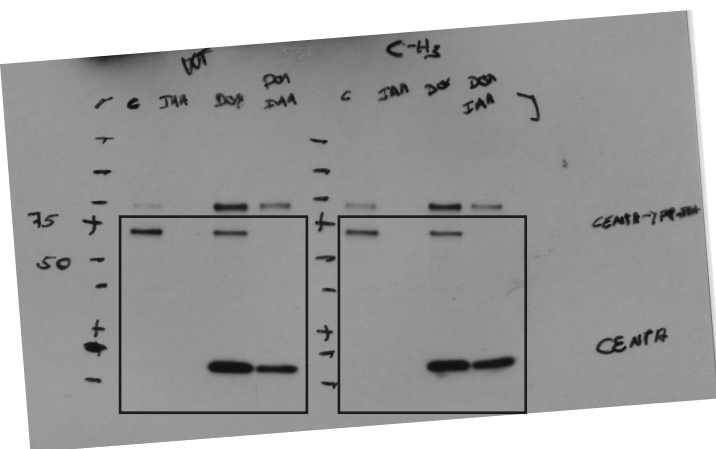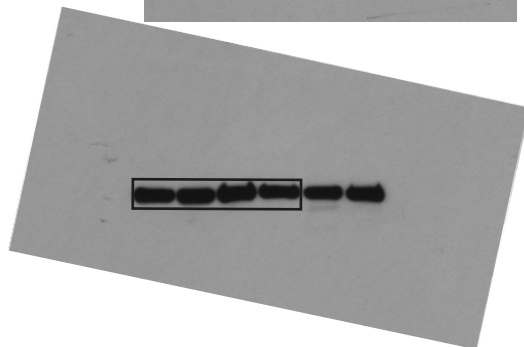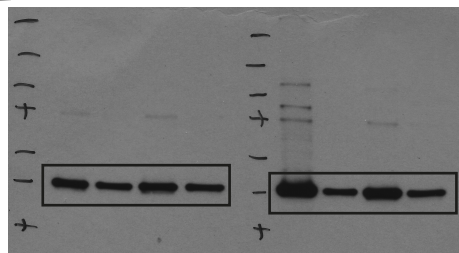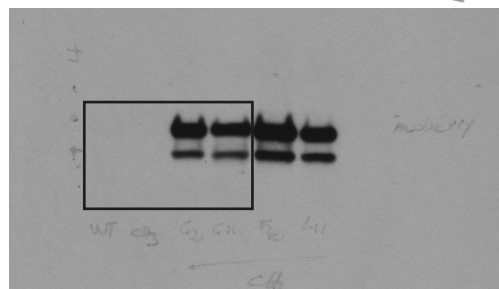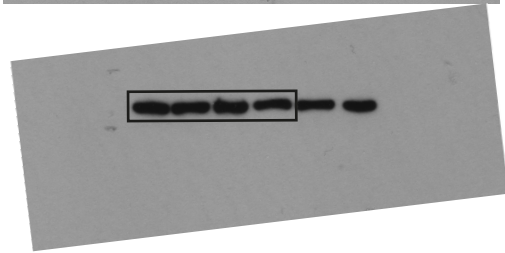

Supplement: SourceData FS1 — is the source file for Fig. S1. [file JCB_202203021_SourceDataFS1.pdf]

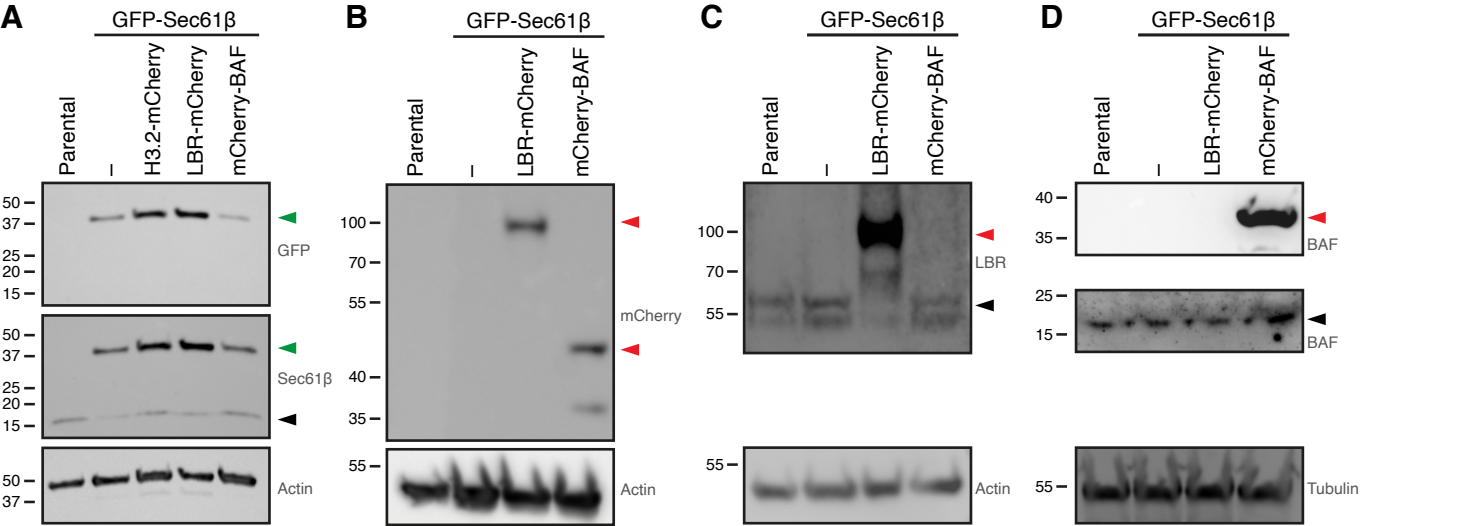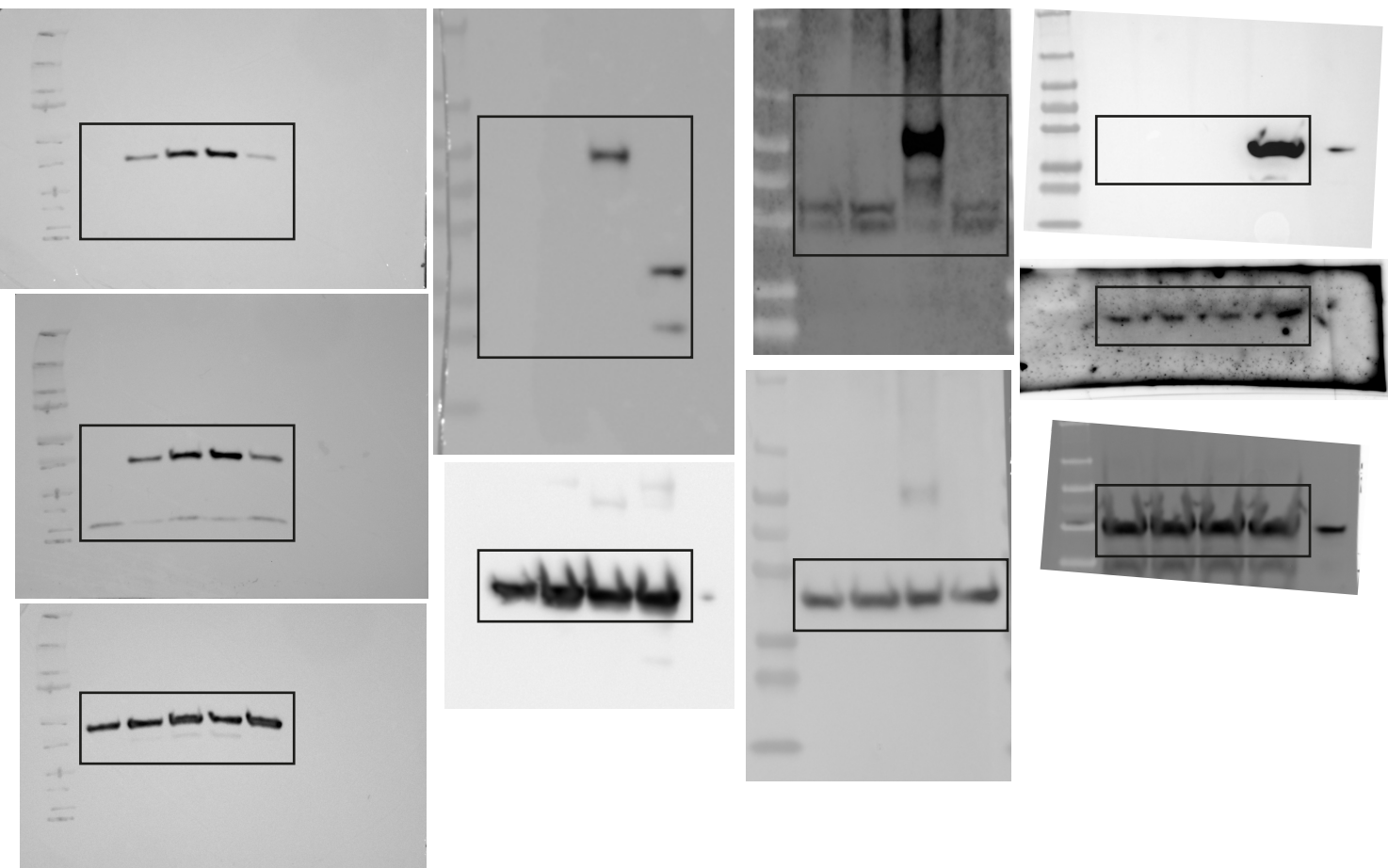

Supplement: SourceData FS5 — is the source file for Fig. S5. [file JCB_202203021_SourceDataFS5.pdf]
